# Research on correlation between English writing self-efficacy and psychological anxiety of college students

**DOI:** 10.3389/fpsyg.2022.957664

**Published:** 2022-07-27

**Authors:** Bai Li

**Affiliations:** School of Foreign Languages, China Pharmaceutical University, Nanjing, China

**Keywords:** English writing self-efficacy, English writing psychological anxiety, college students, English writing motivation, correlation

## Abstract

English writing is not only a cognitive process for college students, it is also influenced by factors related to the emotional or psychological level. With the continuous improvement of the quality of university education cultivation, the pressure faced by college students has increased significantly, which has also given rise to general anxiety among college student groups. In order to investigate the correlation between college students’ English writing self-efficacy and English writing psychological anxiety, we conducted a questionnaire survey on 595 current college students who were not majoring in English at a university in China, and used SPSS 25.0 software to make an empirical analysis of the collected data. The results showed that college students’ English writing self-efficacy was at a moderate level, and they lacked self-confidence in their English writing skills and tended to complete writing tasks; college students generally had a moderate level of psychological anxiety in English writing, among which avoidance behavior was the most significant; college students’ English writing self-efficacy was significantly negatively related to English writing psychological anxiety. English writing self-efficacy can reduce psychological anxiety level by enhancing college students’ English writing motivation. Therefore, we suggest that we should set up English writing courses based on the process teaching method, change college students’ English writing psychology in teaching, set reasonable writing goals, mobilize their enthusiasm and motivation in English writing, reduce college students’ writing anxiety, and enhance their self-confidence in English writing.

## Introduction

With the development and the progress of society over time, English has gradually become a compulsory course for every Chinese student, which has been generally valued by schools and students at all levels. For non-English majors, they face a series of changes in learning requirements and learning patterns from high school English to college English, and are especially prone to the problem of psychological anxiety in English writing. As a basic skill, English writing can not only express the values and emotional attitudes of college students, but also measure their comprehensive quality. According to Mcleod (1987), writing is not only a cognitive composition of English, but also an effective activity that is accompanied by an individual’s emotional engagement. In recent years, more and more attention has been paid to the influence of emotional and psychological factors of college students on English writing. These studies have mainly focused on the relationship between college students’ English writing self-efficacy and their writing level, writing ability, and writing achievement.

According to the latest requirements of Chinese English curriculum standards, one of the goals of English writing teaching is to enhance students’ self-confidence and motivation in English writing. In order to achieve this goal, Chinese colleges and universities have set up corresponding writing tasks in English courses for college students. For example, college students are given writing tasks of practical value (such as invitations, notices, etc.) that they can complete independently, thus improving their writing skills and self-efficacy. Bandura (1977) put forward the concept of self-efficacy, which is defined as the individual’s confidence and belief in his or her ability to complete a task. In 1986, Bandura (1986) went further and pointed out that self-efficacy is not only a judgment of one’s own ability, but also includes expectations of one’s own ability. Combining self-efficacy with English writing gives rise to the concept of English writing self-efficacy. English writing self-efficacy refers to the confidence of English writers in their ability to perform a particular English writing task (Mitchell et al., 2017). Research on English writing self-efficacy points out that English writing self-efficacy can improve an individual’s English writing ability and writing achievement (Murdoch and Kang, 2019).

However, for Chinese non-English majors, writing in English is a very challenging task, which will be affected not only by positive factors (e.g., self-efficacy), but also by negative anxiety (Cheng, 2004; Woodrow, 2011). In the context of English writing, college students’ psychological anxiety is regarded as a kind of trait anxiety, which is a relatively strong fear and avoidance psychology for English Writing (Daly and Wilson, 1983; Woodrow, 2011) and can seriously hinder the improvement of college students’ English writing skills. A large amount of literature and research results confirm that Chinese college students have a relatively low level of English writing, have obvious fear of writing in English, generally lack motivation and self-confidence in writing, and have more obvious writing psychological barriers and anxiety (Woodrow, 2011; Zhang and Guo, 2012; Li et al., 2013).

Although there are many discussions on English writing self-efficacy in the existing literature (Sun et al., 2021; Zhang et al., 2021), most studies have focused on the current situation of English writing among college students and the impact of English writing self-efficacy on improving college students’ English writing performance, and there is lack of sufficient attention and empirical research on the relationship between English writing self-efficacy and psychological anxiety and the mediation of English writing motivation among Chinese college students. Based on this, the current study takes college students in a Chinese university as an entry point and focuses on the current situation of English writing psychological anxiety among Chinese college students, the current situation of Chinese college students’ English writing self-efficacy, the correlation between Chinese college students’ English writing self-efficacy and psychological anxiety, and the effect of English writing motivation in the relationship between English writing self-efficacy and psychological anxiety. By answering these questions, this study hopes to put forward targeted suggestions to alleviate writing psychological anxiety and improving English writing self-efficacy among Chinese college students on the basis of complementing existing research.

## Literature review

### English writing psychological anxiety

Psychological anxiety is an important component of anxiety, which corresponds to physiological anxiety. Generally, psychological anxiety refers to the emotional experience and behavioral performance of an individual in an anxious state. In terms of emotional experience, individuals under psychological anxiety may perceive fear, worry, nervousness, uneasiness, and annoyance, and may even have a sense of panic and imminent death. In terms of behavioral performance, individuals under psychological anxiety may experience fidgeting, facial tension, and sleepless nights (Li et al., 2021; Wang and Zhang, 2021). Focusing on the aspect of English writing, the term psychological anxiety can be extended to writing anxiety or English writing psychological anxiety. The term writing anxiety was first introduced by Daly and Miller in 1975, who argued that writing anxiety is an anxious behavior exhibited by writers in the aggregate of the writing process, such as avoiding writing task and worrying about the content of their writing being read or commented on by others. Furthermore, Daly and Miller (1975) proposed that writing anxiety is a higher level and more intense state of psychological anxiety or psychological fear, not a general state of psychological tension. When writers experience psychological anxiety, their attention to writing is distracted, and the quality of their writing decreases dramatically. Since then, this concept has attracted the attention of many scholars, and these studies cover the conceptual deepening and scale development of writing anxiety (Cheng, 2004), the current situation of foreign language writing anxiety among students in different countries, their coping strategies for writing anxiety (Salam and Al Dyiar, 2014; Torres et al., 2020; Qadir et al., 2021; Abolhasani et al., 2022), and the consequences of writing anxiety (Zhu, 2021). In this study, we no longer distinguishes between psychological anxiety and writing anxiety, but rather in the form of psychological anxiety throughout the text.

### English writing self-efficacy

The concept of English writing self-efficacy is derived from self-efficacy, which was introduced by Bandura (1977), who summarized the influencing results of self-efficacy in four aspects, which are determining the individual’s choice and persistence in a certain activity, affecting the individual’s attitude toward difficulties, affecting the acquisition and subsequent performance of new behaviors, and affecting the individual’s emotion when engaging in a certain activity. Existing research has shown that individuals with this overall confidence are able to make good predictions about the problems they face, and can effectively cope with whatever difficulties they face (Jin et al., 2022). The concept of writing self-efficacy or English writing self-efficacy emerged when self-efficacy was developed in English writing. McCarthy et al. (1985) first put forward the concept of writing self-efficacy, defining it as an individual’s perception and evaluation of his or her own writing skills. Pajares (2003) also combined self-efficacy with writing in his study and proposed a definition of writing self-efficacy, stating that writing self-efficacy is an individual’s self-perceived judgment of the ability to use various writing knowledge and skills to accomplish different writing tasks. Furthermore, he also provided an in-depth analysis of writing self-efficacy, pointing out that writing self-efficacy can significantly promote the improvement of writing achievement, and may also be related to writing anxiety, achievement goals, and expected outcomes. In recent years, writing self-efficacy has received more and more attention, and the research has identified group differences and fluctuations in writing self-efficacy (Mendoza et al., 2022).

### English writing motivation

Motivation is a necessary factor of language learning, the resource that language and language use are equally dependent on. Lowie et al. (2009) claimed that motivation is an adaptable interconnected factor but not a fixed one; high levels of it will lead to learning success. For example, Kormos and Csizér (2008) found that the idea that students’ attitudes toward the role of English in the globalized world are important in English learning. Statistics indicated that Chinese college students vary in their motivation to learn English with variables, such as regional and urban/rural disparities, as well as educational discrepancy (You and Dörnyei, 2016). Writing is difficult with motivational challenges and writing motivation plays a critical role in predicting writers’ performance, shaping learners’ writing experience, and performance (Wright et al., 2019). Previous research showed that more motivated students with past successful experiences in writing are more likely to improve their writing, while college students with low motivation are more likely to procrastinate writing (Hayes, 1996; Fritzsche et al., 2003; Wright et al., 2019).

## English writing self-efficacy and psychological anxiety

In terms of the relationship between English writing self-efficacy and psychological anxiety among college students, the present study holds that there is a negative influence on each other. On the one hand, from the perspective of the differential effects of the two on college students’ English writing performance and writing improvement, English writing-related studies have found that psychological anxiety was significantly and negatively related to writing performance and writing improvement. Students with higher levels of psychological anxiety performed worse in writing performance than those with lower levels of psychological anxiety (Horwitz, 2001; Li et al., 2013). However, the relevant studies focusing on the relationship between writing self-efficacy and writing achievement have reached the opposite conclusion. These studies showed that there was a significant positive correlation between English writing self-efficacy and writing achievement (Pajares, 2003; Woodrow, 2011). Thus, it can be seen that college students’ writing self-efficacy has a negative relationship with psychological anxiety. On the other hand, college students with a high sense of self-efficacy in English writing mean that individuals have high confidence in their ability to successfully complete their writing task. They will be subject to self-reinforcement (observation, feedback, self-reward, etc.) during the English writing process, and this reinforcement effect will directly serve as important motivational factors to enhance college students’ English writing, such as effort, commitment, persistence, strategy use, ability attribution, etc., thus promoting them to better complete the writing task. As a result, they will have less writing anxiety and less writing avoidance behavior, and their psychological anxiety level will naturally decrease (Zeng et al., 2020). Accordingly, the present study suggests that there is a negative relationship between college students’ English writing self-efficacy and psychological anxiety.

Furthermore, college students’ English writing self-efficacy can alleviate their psychological anxiety by enhancing their English writing motivation. Gardner and Lambert (1972) classified learning motivation into instrumental learning motivation and integrative learning motivation. The former refers to learning a language and using it as a tool to achieve a practical purpose, while the latter refers to learning a language and viewing it as an activity to understand and integrate into the culture. However, more research has divided learning motivation into intrinsic motivation and extrinsic motivation. Intrinsic motivation corresponds to integrative motivation, which is to derive pleasure and satisfaction from language learning activities, while extrinsic motivation corresponds to instrumental motivation, which is to take language learning as a way to achieve a certain goal. Existing studies have linked learning motivation with self-evaluation, learning strategies, and psychological anxiety. In the present study, we believe that college students with high self-efficacy in English writing will enhance their writing activity and enthusiasm to participate in English writing, which significantly stimulates their learning motivation, encourages them to put more efforts into English writing, and thus overcoming difficulties and reducing psychological anxiety. Accordingly, we suggests that college students’ English writing motivation plays a mediating role between writing self-efficacy and psychological anxiety.

## Research methodology

### Research questions

Understanding the developmental level of English writing self-efficacy and the status quo of psychological anxiety among Chinese college students, and presenting the relationship between the two, will be of reference and guidance to both the educational activities of Chinese college teachers and the learning activities of college students. The research questions in this research include:

(1) What is the status quo of English writing self-efficacy among Chinese college students?
(2) What is the status quo of English writing psychological anxiety among Chinese college students?
(3) Is there a correlation between English writing self-efficacy and psychological anxiety among Chinese college students? What is the correlation?
(4) Can Chinese college students’ English writing self-efficacy reduce their psychological anxiety by enhancing their writing motivation?

### Participants

In this study, 595 current college students who were not English majors at a Chinese university were randomly selected as the research participants, and four upper and lower grades were also sampled to find out the overall status of English writing self-efficacy and psychological anxiety. There are two reasons for this selection: first, college students in each grade have different English learning times, different English learning strategies, different abilities to use different strategies to complete English writing tasks, and different writing knowledge and writing skills. Therefore, we randomly selects college students from the first to the fourth year of college as the research subjects, which can better enhance the accuracy and comprehensiveness of this study. Second, the selection of college students in four grades as the research subjects can reflect the status quo of English writing self-efficacy and psychological anxiety of Chinese college students more comprehensively, and reflect the overall state and characteristics of English writing of Chinese college students. The research results can better provide a reference for the design of college English writing teaching.

### Instruments

#### English writing self-efficacy questionnaire

This study used the writing self-efficacy scale developed by Jones (2008) to measure the status quo of Chinese college students’ English writing self-efficacy, while we made adjustments to some of the textual expressions in the scale to form the Chinese college students’ English writing self-efficacy scale. This scale includes English writing task self-efficacy and English writing skill self-efficacy. English writing task self-efficacy refers to college students’ judgments about their ability to successfully complete a certain writing task. English writing skill self-efficacy refers to college students’ judgments about their ability to successfully complete various English writing skills. The task self-efficacy subscale has 10 items, which mainly measure general writing tasks and application writing tasks. The sample items are “I believe I can write an email in English to introduce my school to my foreign friends” and “I believe I can write an English composition of at least 80 words within the specified time according to the topic requirements.” The skill self-efficacy sub-scale has 10 items, which mainly measures expressive writing skills, organizational writing skills, stylistic writing skills, and revision writing skills. The sample items are “I believe I can spell all words in an English composition correctly and use punctuation correctly,” “I believe I can finish a writing task within the specified time according to the topic requirements,” “I believe I am good at writing different subjects in English, such as narratives essays, argumentative essays, and letters,” and “I think I can find out the advantages and disadvantages and correct the shortcomings after finishing my English composition.”

#### English writing psychological anxiety questionnaire

The present study used the foreign language writing anxiety scale revised by Guo and Qin (2010) to measure the status quo of English writing psychological anxiety among Chinese college students based on Cheng (2004) study. We adjusted some of the expressions in the original scale to form the Chinese college students’ English writing psychological anxiety scale. This scale includes project teaching anxiety, ideation anxiety, avoidance anxiety, and confidence anxiety. The project teaching anxiety sub-scale has five items, and a sample item is “When I write an English composition, I will feel nervous and uneasy if I know that the teacher will review it.” The ideation anxiety sub-scale has five items, and a sample item is “My mind stops spinning when I am asked to write a time-limited English composition without preparation.” The avoidance anxiety sub-scale has five items, and a sample item is “I usually don’t write in English unless I have no other choice.” The confidence anxiety sub-scale has five items, and a sample item is “I will worry about getting a very low score when my English composition is reviewed.”

#### English writing motivation questionnaire

This study modified and adjusted some of the expressions to form the Chinese college students’ English writing motivation scale based on the English learning motivation questionnaire developed by Gao et al. (2003). This scale includes two sub-scales: intrinsic motivation sub-scale and extrinsic motivation sub-scale. The intrinsic writing motivation scale has seven items, and a sample item is “The reason why I finish my English writing is my love for English.” The extrinsic writing motivation scale has 15 items, and a sample item is “The reason why I finish my English writing is to get good grades so as to get a good job.”

#### Reliability and validity tests

We examined the reliability and validity of the Chinese college students’ English writing self-efficacy scale, the Chinese college students’ psychological anxiety scale, and the Chinese college students’ English writing motivation scale. Unless otherwise noted, responses to all items were measured on five-point Likert-type scales.

The Chinese college students’ English writing self-efficacy scale consists of 20 items, ranged from strongly disagree (1) to strongly agree (5). The mean score of all 20 items was the overall English writing self-efficacy level of college students. We conducted a confirmatory factor analysis on the whole scale. The KMO value was 0.890, the Bartlett’s sphere test reached a significant level (*p* < 0.001), and the factors with characteristics greater than 1 explained 78.961% of the total variance. Exploratory factor analysis extracted two factors, namely, the English writing task self-efficacy sub-scale and the English writing skill self-efficacy sub-scale. The internal consistency coefficient of the total scale was 0.853, the internal consistency coefficient of factor 1 was 0.901, and the internal consistency coefficient of factor 2 was 0.804. Accordingly, the Chinese college students’ English writing self-efficacy scale had high reliability and internal validity consistency, and was suitable to be used as a research tool for the English writing self-efficacy of Chinese college students.

The Chinese college students’ English writing anxiety scale consists of 20 items, ranged from strongly disagree (1) to strongly agree (5). The mean score of all 20 items was the overall English writing psychological anxiety level of college students. We conducted a confirmatory factor analysis on the whole scale. The KMO value was 0.911, the Bartlett’s sphere test reached a significant level (*p* < 0.001), and the factors with characteristics greater that 1 explained 80.738% of the total variance. Exploratory factor analysis extracted four factors, namely, the English writing project anxiety sub-scale, the English writing ideation anxiety sub-scale, the English writing avoidance anxiety sub-scale, and the English writing confidence anxiety sub-scale. The internal consistency coefficient of the total scale was 0.883, the internal consistency coefficient of factor 1 was 0.897, the internal consistency coefficient of factor 2 was 0.865, the internal consistency coefficient of factor 3 was 0.937, and the internal consistency coefficient of factor 1 was 0.900. Accordingly, the Chinese college students’ English writing psychological anxiety scale had high consistency reliability and internal validity, and was suitable to be used as a research tool for English writing psychological anxiety of Chinese college students.

The Chinese college students’ English writing motivation scale consists of 22 items, ranged from strongly disagree (1) to strongly agree (5). The mean score of all 22 items was the overall English writing motivation level of college students. We conducted a confirmatory factor analysis on the whole scale. The KMO value was 0.934, the Bartlett’s sphere test reached a significant level (*p* < 0.001), and the factors with characteristics greater that 1 explained 82.974% of the total variance. Exploratory factor analysis extracted two factors, namely, the English writing intrinsic motivation sub-scale and the English writing extrinsic motivation sub-scale. The internal consistency coefficient of the total scale was 0.874, the internal consistency coefficient of factor 1 was 0.869, and the internal consistency coefficient of factor 2 was 0.883. Accordingly, the Chinese college students’ English writing motivation scale had high reliability and internal validity consistency, and was suitable to be used as a research tool for the English writing motivation of Chinese college students.

### Questionnaire distribution and data analysis

A questionnaire survey was conducted among college students in a Chinese university at the beginning of March 2022. In order to ensure the validity of the data, an electronic version of the questionnaire was sent to each college student after confirming that they fully understood the purpose and relevant requirements of the survey. A total of 700 surveys were distributed and 650 surveys were collected, of which 595 were effective, with an effective response rate of 85%.

We used SPSS 25.0 for data analysis. First, descriptive statistical analysis was applied to investigate the development level of college students’ English writing self-efficacy and the status quo of college students; English writing psychological anxiety. Second, correlation analysis was applied to verify the correlation between English writing self-efficacy and psychological anxiety among college students. Finally, structural equation modeling (SEM) was used to test the mediating role of English writing motivation between college students’ English writing self-efficacy and psychological anxiety.

## Results and discussion

### The status quo of college students’ English writing self-efficacy

As shown in Table 1, the results of descriptive statistical analysis of Chinese college students’ English writing self-efficacy show that the scores of college students’ overall English writing self-efficacy (*M* = 3.0619, SD = 0.5978), English writing task self-efficacy (*M* = 3.1250, SD = 0.6184), and English writing skill self-efficacy (*M* = 2.9987, SD = 0.6033) are not high, and all lower than 3.5. According to Pajares (1996), the mean value of English writing self-efficacy is low between 1.0 and 1.5, medium between 1.5 and 3.5, and high between 3.5 and 5.0. It can be seen that Chinese college students’ English writing self-efficacy is at a moderate level. Comparing the means of English writing task self-efficacy with skill self-efficacy, we can find that the mean of English writing task self-efficacy is higher than that of skill self-efficacy, which indicates that Chinese college students are essentially more confident in English writing tasks despite the fact that they have mastered certain English writing skills after years of writing practice. It also means that Chinese college students are more inclined to regard English writing as a task and lack confidence in their own English skills. In addition, the SD of college students’ overall English writing self-efficacy, English writing task self-efficacy, and English writing skill self-efficacy was less than 1, which indicates that although the overall level of English writing self-efficacy among Chinese college students is not high, the development level is relatively balanced.

**TABLE 1:** Results of descriptive analysis of English writing self-efficacy.

	*N*	Item	Min	Max	Mean	Standard deviation
English writing self-efficacy (EWS)	595	20	1.26	4.86	3.0619	0.5978
English writing task self-efficacy (EWTS)	595	10	1.27	4.98	3.1250	0.6184
English writing skill self-efficacy (EWSS)	595	10	1.19	4.81	2.9987	0.6233

Furthermore, this study conducted a descriptive analysis of each relevant dimension of college students’ English writing self-efficacy. Writing task self-efficacy includes general writing task self-efficacy and applied writing task self-efficacy. General English writing task self-efficacy refers to college students’ judgment of their ability to complete general writing tasks, while applied writing task self-efficacy refers to college students’ judgment of their ability to complete practical writing tasks. The mean of general writing task self-efficacy is 3.1528, and the mean of applied writing task self-efficacy is 3.3325, indicating that Chinese college students are more confident in the task of applied writing category, which may be related to the fact that college students pay more attention to the practical value of English writing more and have received more training in applied writing. For example, college students generally score higher in writing invitation letters and introduction letters. Writing skill self-efficacy includes four aspects: expressive writing, organizational writing, genre writing, and revision writing. The mean of expressive writing skill self-efficacy is significantly higher than the other three, which indicates that Chinese college students show more confidence in test-taking abilities, such as spelling words and using punctuation marks correctly, judging wordiness, and quickly examining questions. It may be related to their college entrance examination experience. According to the above clues, college teachers can select materials appropriately and flexibly when teaching English writing, which is not limited to the topics with high applicability, but also can be broaden to more general genres, so as to promote the all-round development of college students’ English writing ability while maintaining their self-confidence in application-based essays.

### The status quo of college students’ English writing psychological anxiety

As shown in Table 2, the results of descriptive statistical analysis of Chinese college students’ English writing psychological anxiety show that except for project teaching anxiety (*M* = 2.7912, SD = 0.7862) scored low, college students’ overall English writing psychological anxiety (*M* = 3.2852, SD = 0.6831), English writing ideation anxiety (*M* = 3.0313, SD = 0.8277), English writing avoidance anxiety (*M* = 3.7745, SD = 0.5154), and English writing confidence anxiety (*M* = 3.5438, SD = 0.6396) scored higher 3.0. English writing avoidance anxiety scored the highest with over 3.5. This shows that Chinese college students’ English writing anxiety is generally at a moderate level. Despite the fact that they have mastered certain English writing skills after many years of writing practice, Chinese college students are still essentially more confident in English writing tasks. It also indicates that Chinese college students tend to see English writing as a task and lack confidence in their own English skills.

**TABLE 2:** Results of descriptive analysis of English writing psychological anxiety.

	*N*	Item	Min	Max	Mean	Standard deviation
English writing psychological anxiety (EWPA)	595	20	1	5	3.2852	0.6831
English writing project teaching anxiety (EWPTA)	595	5	1	5	2.7912	0.7862
English writing ideation anxiety (EWIA)	595	5	1	5	3.0313	0.8277
English writing avoidance anxiety (EWAA)	595	5	2.23	5	3.7745	0.5154
English writing confidence anxiety (EWCA)	595	5	1.63	5	3.5438	0.6396

We further conducted a descriptive analysis of each relevant dimension of college students’ psychological anxiety. Among the four dimensions of English writing psychological anxiety, Chinese college students’ avoidance anxiety is particularly serious, followed by confidence anxiety, while project teaching anxiety is relatively less serious, indicating that Chinese college students show high levels of anxiety in English writing avoidance anxiety and confidence anxiety. Facing English writing, most college students choose to avoid and lack confidence in their English writing, which may be due to the fact that Chinese college students have less daily exposure to the topic of English writing and are not interested in English writing. This enlightens that teachers in university need to reduce the assignment of writing practice tasks during English writing classes, interact more with students, and enhance college students’ interest in English writing, while focusing on college students’ progress in the process of English writing rather than only on their English writing scores. Project teaching anxiety and ideation anxiety belong to low-anxiety levels, and the possibility of triggering college students’ anxiety is relatively small. However, in our further analysis, we also found that college students still experience writing stress, nervousness, and blankness, especially when they are faced with the requirement to complete the English writing tasks within the specified time. Project teaching anxiety mainly occurs when college students are unable to respond to the teacher’s questions. It is an anxiety phenomenon arising from the protection of self-image. This suggests that teachers in university should relax time as much as possible when assigning writing tasks. They also can set up study groups to give students the opportunity to brainstorm before writing, and ask divergent questions to encourage students to dare to answer, rather than focusing on whether the answers are correct.

### Correlation between college students’ English writing self-efficacy and psychological anxiety

To further understand the relationship between Chinese college students’ English writing self-efficacy and psychological anxiety, the present study used the correlation coefficient to analyze the correlation between English writing task self-efficacy, skill self-efficacy, and four types of psychological anxiety.

As shown in Table 3, the simple correlation coefficients between English writing self-efficacy and psychological anxiety was −0.287. The two dimensions of English writing self-efficacy and psychological anxiety were −0.269 and −0.301, respectively. These results indicated that there was a negative correlation between English writing self-efficacy and psychological anxiety. The higher the college students’ English writing efficacy, the lower the level of psychological anxiety. Furthermore, we also calculated the correlations between different dimensions of English writing self-efficacy and different dimensions of psychological anxiety, and the results showed that there was a significant negative between different dimensions of both.

**TABLE 3:** Results of the correlation analysis between English writing self-efficacy and psychological anxiety.

		EWPA	EWPTA	EWIA	EWAA	EWCA
EWS	*r*	−0.287**	−0.257**	−0.266**	−0.283**	−0.300**
EWTS	*r*	−0.269**	−0.191*	−0.200**	−0.336**	−0.295**
EWSS	*r*	−0.301**	−0.244**	−0.276**	−0.332**	−0.299**

**p < 0.01, *p < 0.05.

We applied a combination of path analysis and SEM to verify the mediating role of English writing motivation in the relationship between English writing self-efficacy and psychological anxiety among college students. First, the predictive effect of English writing self-efficacy on psychological anxiety was examined. According to the results of path analysis, the standardized coefficient between English writing self-efficacy and psychological anxiety was −0.229, with a standard error of 0.064, *t* = −4.138, and a significance level of *p* = 0.000 (<0.001), which indicates that writing self-efficacy has a significant impact on psychological anxiety, and for each unit increase in writing self-efficacy one unit, psychological anxiety decreased by 0.229 units.

**FIGURE 1:**
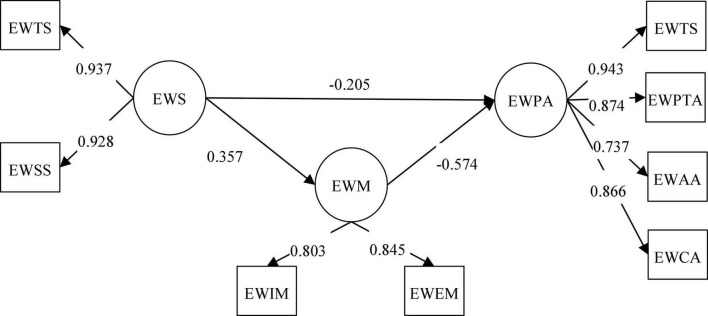
Results of the mediating effect of writing motivation.

We applied SEM to examine the mediating effect of writing motivation. The results showed that theoretical model (χ^2^/df = 3.025, RMSEA = 0.060, CFI = 0.917, TLI = 0.905, SRMR = 0.058) fitted the data well. Meanwhile, the two dimensions loads of writing self-efficacy were above 0.920, the four dimensions loads of psychological anxiety were between 0.700 and 0.950, and the two dimensions loads of writing motivation were above 0.800 (see Figure 1). In terms of the standardized coefficients, the effect of writing self-efficacy on writing motivation was significantly positive (β = 0.357, *p* < 0.001), the effect of writing motivation on psychological anxiety was significantly negative (β = −0.574, *p* < 0.001), and the effect of writing self-efficacy on psychological anxiety was still significantly negative (β = −0.205, *p* < 0.001), but the standardized coefficient was smaller. Thus, college students’ writing motivation plays a partial mediating role in the relationship between writing self-efficacy and psychological anxiety. Writing self-efficacy can directly reduce college students’ psychological anxiety, and can also alleviate psychological anxiety by enhancing their writing motivation.

## Conclusion and implication

### Research conclusion

The present study found that college students’ overall English writing self-efficacy was at a moderate level, and Chinese college students lacked self-confidence in their English writing skills and tended to complete writing tasks. They generally had a moderate level of English writing psychological anxiety, with avoidance behavior being the most significant. College students’ writing self-efficacy was significantly negatively related to psychological anxiety, and writing self-efficacy could reduce psychological anxiety by enhancing their writing motivation. The results reaffirm the previous literature on the negative effect of self-efficacy on psychological anxiety in the context of Chinese college students’ English writing.

### Practical implications

Our study provides pedagogical recommendations based on the research findings. First, enhance college students’ English writing self-efficacy and cultivate their interest in English writing. According to the findings in our study, Chinese college students’ English writing self-efficacy level is not high, i.e., they are not confident enough in English writing. Thus, we suggest that college teachers need to pay attention to enhancing college students’ self-efficacy in English writing in the teaching process (Bandura, 1977). For example, they should consolidate the basic knowledge of the English language and improve the language foundation of college students. When reviewing college students’ English writing, the teacher should not only focus on word or grammatical errors, but also pay attention to the outstanding points of college students’ English writing and give them positive and encouraging comments. In these ways, college students’ self-confidence in English writing can be improved. In terms of cultivating college students’ interest in English writing, the reason for this phenomenon that college students have a high level of English writing avoidance anxiety is the lack of interest, which means that college teachers should consciously cultivate college students’ interest. In the topic selection, they should choose those writing topics that are close to the daily life of college students to reduce their avoidance psychology. In the review of writing, they should correct the English writing submitted by college students in time and give them timely feedback to enhance their learning motivation.

Second, pay attention to college students’ English writing psychological anxiety. According to the high level of psychological anxiety among Chinese college students found in our study and the negative relationship between English writing self-efficacy and psychological anxiety, college teachers should pay attention to college students’ attitudes toward English writing in their usual English writing teaching, actively communicate with students, and understand their emotional expressions. For example, when observing the anxiety of college students, teachers should take the initiative to communicate with them and tell them about the normality of anxiety to weaken the psychological burden of college students. Besides, one important reason for the emergence of English writing psychological anxiety is the difficulties encountered in English writing. Pre-writing inspiration and conception or pre-writing preparation activities for college students can help to reduce their English writing psychological anxiety. In order to enrich college students’ language knowledge and improve their language output skills, colleges teachers should increase comprehensible input as much as possible in the training of comprehensive language skills. At the same time, with a certain language’s skills as a guarantee, college teachers should try to provide college students with various themes, and interesting content in writing materials. They also should assign different genres of writing tasks and try different forms of writing, such as poem, prose, and novel. College students should ensure enough writing output and improve the quality of written English. The improvement of writing ability is the basic way to eliminate English writing psychological anxiety.

Finally, strengthen the English writing motivation training of college students. Enhancing English writing motivation can also alleviate psychological anxiety for college students. Teachers in universities can apply the process writing teaching method in teaching, and carry out a series of idea training, such as brainstorming, to increase students’ writing ideas before they write in English, so as to relieve the psychological anxiety caused by the lack of ideas (Rusinovci, 2015). In order to change college teachers’ traditional evaluation method, we think teachers in colleges should reduce error-correcting composition and pay more attention to college students’ writing process. This means that teachers in colleges need to shift students’ attention from writing scores to the writing process. In the process of writing, what college students get and what wonderful sentences they accumulate in their brain may be more important, not just the numbers given by teachers to represent their writing ability by scores. In fact, the teacher can adopt self-evaluation, peer evaluation, and group evaluation to evaluate students’ English writing.

### Limitations and suggestions

Although the present study achieves the research aims and obtains some valuable findings, our study still has some limitations. As Pajares and Valiante (2012) mentioned in their study, exploring causal relationships between variables in non-experimental contexts requires sound theoretical assumptions about the model and considerable caution in interpreting the results. They also suggested that different theoretical models could be tested with more powerful statistical tools, such as SEM. Although we used SEM to suggest the positive effect of English writing self-efficacy on relieving psychological anxiety and the mediating effect of writing motivation in the relationship from the theoretical and existing findings on English writing self-efficacy, English writing psychological anxiety, and writing motivation, and these findings were tested among Chinese college students, the results still could not clarify in the cross-sectional study. In the follow-up study, we can use the tracking research paradigm to explore the deeper causal relationships. In addition, our study is a sample from a single university, with no mention of the University courses involved, so its results are limited in terms of generalization.

## Data availability statement

The raw data supporting the conclusions of this article will be made available by the authors, without undue reservation.

## Ethics statement

Ethical review and approval was not required for the study on human participants in accordance with the local legislation and institutional requirements. Written informed consent from the patients/participants or patients/participants legal guardian/next of kin was not required to participate in this study in accordance with the national legislation and the institutional requirements.

## Author contributions

BL made significant contributions to the study concept and design. He was primarily responsible for designing the study, collecting and analyzing data, and drafting the manuscript. Also, he made several revisions and refinements to the content of the manuscript.
